# Association of stress hyperglycemia ratio and poor long-term prognosis in patients with myocardial infarction with non-obstructive coronary arteries

**DOI:** 10.1186/s12933-023-01742-6

**Published:** 2023-01-16

**Authors:** Fuad A. Abdu, Jassur Galip, Penglong Qi, Wen Zhang, Abdul-Quddus Mohammed, Lu Liu, Guoqing Yin, Ayman A. Mohammed, Redhwan M. Mareai, Rong Jiang, Yawei Xu, Wenliang Che

**Affiliations:** 1 Department of Cardiology, Shanghai Tenth People’s Hospital, Tongji University School of Medicine, 301 Yanchang Road, Shanghai, 200072 China; 2 Department of Cardiology, Shanghai Tenth People’s Hospital Chongming branch, Shanghai, China

**Keywords:** Myocardial infarction with non-obstructive coronary arteries, Stress hyperglycemia ratio, Diabetes mellitus, Clinical outcome

## Abstract

**Background:**

Stress hyperglycemia ratio (SHR) is a novel biomarker of true acute hyperglycemia condition and is associated with a worse prognosis in patients with myocardial infarction (MI). However, the effects of SHR in the setting of MI with non-obstructive coronary arteries (MINOCA) have not been investigated. This study aimed to explore the association between SHR and long-term clinical outcomes among MINOCA patients.

**Methods:**

A total of 410 MINOCA patients were included in the final analysis of this study. The patients were divided into three groups based on the SHR tertiles: [SHR1 group (SHR ≤ 0.73), (n = 143); SHR2 group (SHR 0.73–0.84), n = 131; and SHR3 group (SHR ≥ 0.84), n = 136]. Follow-up for major adverse cardiovascular events (MACE) was conducted on all patients. Cox regression and Kaplan–Meier curve analysis were used to evaluate the relationship between SHR and MACE. The receiver operating curve (ROC) analysis was applied to obtain the optimal cut-off value of SHR for predicting clinical MACE.

**Results:**

A total of 92 patients developed MACE during the mean 34 months of follow-up. A significant increase in MACE was observed in the SHR3 group compared to the SHR1 and SHR2 groups (35.3% vs. 15.4% and 16.8%, respectively; P < 0.001). The Kaplan–Meier curves demonstrate that SHR3 patients had the highest MACE risk compared to SHR1 and SHR2 patients (log-rank P < 0.001). In addition, when both SHR tertiles and diabetes status were considered, those with SHR3 and diabetes had the highest hazard of MACE (log-rank P < 0.001). Multivariate Cox regression analysis showed that the SHR3 is associated with a 2.465-fold increase in the risk of MACE (adjusted HR, 2.465; 95% CI 1.461–4.159, P = 0.001). The ROC curve analysis showed that the optimal SHR cut-off value for predicting clinical MACE among MINOCA was 0.86.

**Conclusion:**

Our data indicates, for the first time, that SHR is independently associated with poor long-term prognosis in patients suffering from MINOCA. The optimal SHR cut-off value for predicting clinical MACE among MINOCA patients was 0.86. These findings suggest that SHR may play a potential role in the cardiovascular risk stratification of the MINOCA population.

**Supplementary Information:**

The online version contains supplementary material available at 10.1186/s12933-023-01742-6.

## Background

Cardiovascular diseases (CVD), particularly acute myocardial infarction (AMI), remain a growing threat to public health and a leading cause of morbidity and mortality worldwide [1]. Myocardial infarction with non-obstructive coronary arteries (MINOCA) is a common clinical condition observed in around 5–10% of all patients with AMI admitted for coronary angiography (CAG) [2, 3]. MINOCA represents a heterogeneous and largely unexplored clinical syndrome with various underlying pathophysiological mechanisms that warrant further investigations [4]. It often remains a misdiagnosed and mismanaged illness linked to a high incidence of major adverse cardiovascular events (MACE), mortality, and a lower quality of life [5, 6]. Previous studies reported that MINOCA is associated with an approximately 23.9% rate of MACE after 4 years of follow-up, and the all-cause mortality rate of MINOCA patients at 1 year and 3 years were 10.9% and 16.1%, respectively [7, 8]. Therefore, it is essential to identify easily obtainable determinant factors of adverse events to provide optimal management and improve the quality of life in this patient population.

Stress hyperglycemia refers to the acute transient increase in blood glucose levels in response to numerous critical conditions and is independently associated with poor short and long-term clinical outcomes in acute coronary syndrome (ACS) patients, particularly those with AMI [9–11]. It has recently been revealed that admission stress hyperglycemia is also common among MINOCA patients [12], and it is a strong predictor of short- and long-term adverse outcomes in this patient group, regardless of diabetes status [13]. Additionally, we and others have recently shown that fasting blood glucose [14], and triglyceride-glucose index [15] were associated with poor clinical outcomes among patients suffering from MINOCA. However, elevated glucose levels at the time of hospital admission may be the result of chronic hyperglycemia or acute stress response [16]. In this regard, the stress hyperglycemia ratio (SHR) has been developed as a new marker to reflect true acute hyperglycemia condition, which is estimated based on the acute admission glucose level and the chronic glycemic value [calculated by glycosylated hemoglobin (HbA1c)] [17]. Several clinical studies have reported that SHR is associated with significantly higher in-hospital mortality and long-term MACE than admission glucose in patients with AMI [16, 18–22]. As of yet, there is no data regarding the impact of SHR on the clinical outcomes among MINOCA patients.

As such, this study sought to explore for the first time the predictive value of SHR and obtain its optimal cut-off value in predicting long-term clinical outcomes among patients suffering from MINOCA and further determine whether it may have any clinical relevance in this population.

## Materials and methods

### Study design and population

During the period 2014 through 2022, we conducted an observational retrospective study of patients with AMI who underwent CAG and had new-onset chest pain with ST-segment elevation MI (STEMI) and non-ST segment elevation MI (NSTEMI) on ECG presenting at the cardiology department of Shanghai Tenth People’s Hospital (Tongji University, Shanghai, China). In this study, MINOCA was defined as patients with evidence of AMI with non-obstructive coronary arteries (defined as stenosis less than 50% in any epicardial coronary arteries), as recommended by the 4th universal definition of AMI [23], which excluded myocarditis and Takotsubo syndrome from the final diagnosis of MINOCA. The exclusion criteria included the following items: (1) patients < 18 years; (2) patients with a history of MI or obstructive CAD; (3) patients receiving thrombolytic prior to or during hospitalization, (4) patients with type 3–5 MI; (5) those with severe liver and kidney conditions; (6) patients with major valve pathologies, a history of stroke, and malignant arrhythmias; and (7) patients lost to follow-up or had no complete SHR data.

Our study was approved by the Shanghai Tenth People’s Hospital ethics committee and complied with the Declaration of Helsinki. Informed consent has been obtained from all patients.

### Data collection and definitions

We retrospectively gathered the baseline demographics (age, gender, height, weight, body mass index (BMI), heart rate, and blood pressure), past medical history (history of hypertension, diabetes, hyperlipidemia, atrial fibrillation, and smoking history), electrocardiogram, and echocardiography information for all patients. Blood samples for testing HbA1c, blood glucose, cardiac troponin-T (cTnT), N-terminal pro-brain natriuretic peptide (NT-proBNP), creatine kinase-MB (CK-MB), total cholesterol (TC), low-density lipoprotein cholesterol (LDL-C), high-density lipoprotein cholesterol (HDL-C), triglyceride (TG), C reactive protein (CRP), and complete blood count (white blood cell counts, red blood cell counts, and hemoglobin) was obtained from the cubital vein after at least eight hours of fasting. An Abbott Laboratories (Chicago, IL, USA) was used to analyze blood glucose, TC, LDL-C, HDL-C, and TG. A diabetes diagnosis is based on the following: (1) Random plasma glucose ≥ 11.1 mmol/l (≥ 200 mg/dl); (2) fasting blood glucose ≥ 7.0 mmol/l (≥ 126 mg/dl); (3) HbA1c ≥ 6.5%; and (4) OGTT glucose level ≥ 11.1 mmol/l (200 mg/dl).

### Determination of SHR

The blood glucose obtained during the first 24 h of hospital admission was considered admission blood glucose. Abbott Laboratories (Chicago, IL, USA) was used to calculate the HbA1c. The SHR is calculated using the following equation by dividing admission glucose by the average glucose calculated from HbA1c: SHR = [(admission glucose (mmol/L) / [1.59 × HbA1c (%) − 2.59] [18].

### Endpoints and follow up

In this study, the mean follow-up duration was 34 months. Clinical outcomes were recorded by two experts via telephone calls, clinic visits, review of medical case history, and communication with patients’ families. The primary observational clinical endpoints of the present investigation were MACE, which includes cardiac death, heart failure, nonfatal MI, stroke, and angina rehospitalization. Deaths caused by malignant arrhythmias, acute MI, heart failure, or other cardiac conditions were defined as cardiac deaths. Nonfatal MI was defined as positive cardiac biomarkers or dynamic changes on electrocardiograms in addition to the typical symptoms of myocardial ischemia. A heart failure diagnosis was made based on recent ESC guidelines for the diagnosis and treatment of acute and chronic heart failure [24]. A stroke is diagnosed when there is evidence of ischemic cerebral infarction because of thrombotic or embolic obstruction.

### Statistical analysis

Statistical Package for the Social Sciences (SPSS) version 24 was used to analyze our data. GraphPad software version 8.0.1 was used to create the figures. We expressed continuous variables as means and standard deviations (mean ± SD), while categorical variables as percentages (%). The comparison of clinical data between groups was made using ANOVAs for continuous variables and Pearson chi-square tests or Fisher’s exact tests for categorical variables. The Kaplan–Meier curve was used to determine the cumulative incidence of clinical outcomes, and a log-rank test was used to determine differences between groups. A Pearson correlation analysis was performed to determine the correlation between SHR and myocardial injury parameters. Univariate Cox regression was used to evaluate the association between SHR and clinical outcomes. Cardiovascular risk factors listed in Table 1 (age, sex, BMI, LVEF, hypertension, diabetes, hyperlipidemia, smoking, atrial fibrillation, STEMI, degree of coronary stenosis, cTnT, CK-MB, NT-proBNP, TC, LDL-C, HDL-C, TG, and CRP) which may contribute to an elevated risk of adverse outcomes among MINOCA patients served as the variables in the univariate analysis along with SHR index. Clinical covariates that were significant with a P < 0.10 in the univariate analysis were used for adjustment in the multivariate analysis by the forward stepwise regression method. The receiver operating curve (ROC) was applied to calculate the area under the curve (AUC) and obtained the optimal cut-off value of SHR for predicting clinical outcomes among MINOCA patients, and the Youden index was calculated at the point where the sensitivity and specificity sum was highest. All analysis was conducted two-sided, and statistical significance was set at P-value < 0.05.

**Table 1:** Clinical characteristics according to different SHR tertiles

Variables	SHR1 (≤ 0.73) N = 143	SHR2 (0.73–0.84) N = 131	SHR3 (≥ 0.84) N = 136	P value
Age (years)	64.05 ± 13.45	63.32 ± 13.45	63.22 ± 14.58	0.860
Male, n (%)	72 (50.3)	73 (55.7)	71 (52.2)	0.667
BMI (kg/m2)	24.13 ± 4.20	24.26 ± 3.15	24.28 ± 4.23	0.970
Comorbidities				
Hypertension, n (%)	70 (49.0)	69 (52.7)	76 (55.9)	0.510
Diabetes, n (%)	28 (19.6)	23 (17.6)	28 (20.6)	0.816
Smoking history, n (%)	57 (39.9)	51 (38.9)	64 (47.1)	0.332
Atrial fibrillation, n (%)	20 (14.0)	8 (6.1)	21 (15.4)	0.041
Hyperlipidaemia, n (%)	19 (13.3)	21 (16.0)	13 (9.6)	0.285
STEMI, n (%)	45 (31.5)	44 (33.6)	39 (28.7)	0.685
Angiographic characteristics				
Normal vessels^a^, n (%)	67 (46.9)	62 (47.3)	61 (44.9)	0.910
Vessel with any stenosis^b^, n (%)	76 (53.1)	69 (52.7)	75 (55.1)	0.910
Echocardiography parameters				
LVEF (%)	55.68 ± 9.82	55.78 ± 10.22	52.73 ± 12.34	0.041
LAD (mm)	37.00 ± 6.53	36.74 ± 6.46	38.71 ± 6.12	0.030
E/e’	10.86 ± 3.20	10.43 ± 2.82	10.18 ± 2.88	0.334
LVEDD (mm)	44.72 ± 5.10	45.30 ± 5.45	47.10 ± 5.97	0.001
LVESD (mm)	29.73 ± 6.07	30.65 ± 12.06	32.03 ± 7.36	0.116
TTPG (mmHg)	24.74 ± 12.74	25.24 ± 7.77	23.68 ± 6.34	0.623
Laboratory parameters				
HbA1c (%)	6.51 ± 1.20	6.35 ± 1.46	6.01 ± 1.23	0.005
FBG (mmol/L)	5.13 ± 1.35	5.90 ± 1.81	7.30 ± 3.20	< 0.001
cTnT (ng/mL)	0.42 ± 1.06	0.42 ± 1.04	1.02 ± 3.15	0.018
Creatine kinase-MB (ng/mL)	14.89 ± 33.52	20.11 ± 53.70	24.54 ± 42.26	0.182
NT-proBNP (pg/mL)	1477.69 ± 3573.61	4639.57 ± 405.36	6541.46 ± 560.92	0.007
TC (mmol/L)	4.27 ± 1.09	4.15 ± 0.93	4.20 ± 1.12	0.675
LDL-C (mmol/L)	2.50 ± 0.92	2.41 ± 0.84	2.43 ± 0.94	0.677
HDL-C (mmol/L)	1.14 ± 0.33	1.14 ± 0.31	1.19 ± 0.37	0.445
TG (mmol/L)	1.52 ± 1.17	1.54 ± 0.93	1.48 ± 0.98	0.873
CRP (mg/dL)	0.68 ± 0.62	0.60 ± 0.87	0.95 ± 1.34	0.012
WBC (10^9^/L)	7.90 ± 2.96	7.89 ± 3.24	8.52 ± 3.74	0.208
RBC (10^ 12^/L)	4.34 ± 0.67	4.42 ± 0.64	4.49 ± 0.60	0.163
Hemoglobin (g/L)	131.69 ± 23.93	133.19 ± 20.32	134.25 ± 18.08	0.599

*SHR* stress hyperglycemia ratio, *BMI* body mass index, *LVEF* left ventricular ejection fraction, *LAD* left atrium diameter, *E/e’* mean septal velocity, *LVEDD* left ventricular end-diastolic diameter, *LVESD* left ventricular end-systolic diameter, *TTPG* trans tricuspid pressure gradient, *STEMI* ST-segment elevation myocardial infarction, *HbA1c* hemoglobin A1c, *FBG* fasting blood glucose, *cTnT* cardiac troponin, *NT-proBNP* N-terminal pro-brain natriuretic peptide, *TC* total cholesterol, *HDL-C* high density lipoprotein, *LDL-C* low-density lipoprotein, *TG* triglyceride, *CRP* C reactive protein, *WBC* white blood cell counts, *RBC* red blood cell

^a^Vessels with 0% stenosis

^b^Vessels with 0–50% stenosis

## Results

### Baseline characteristics

**Fig. 1:**
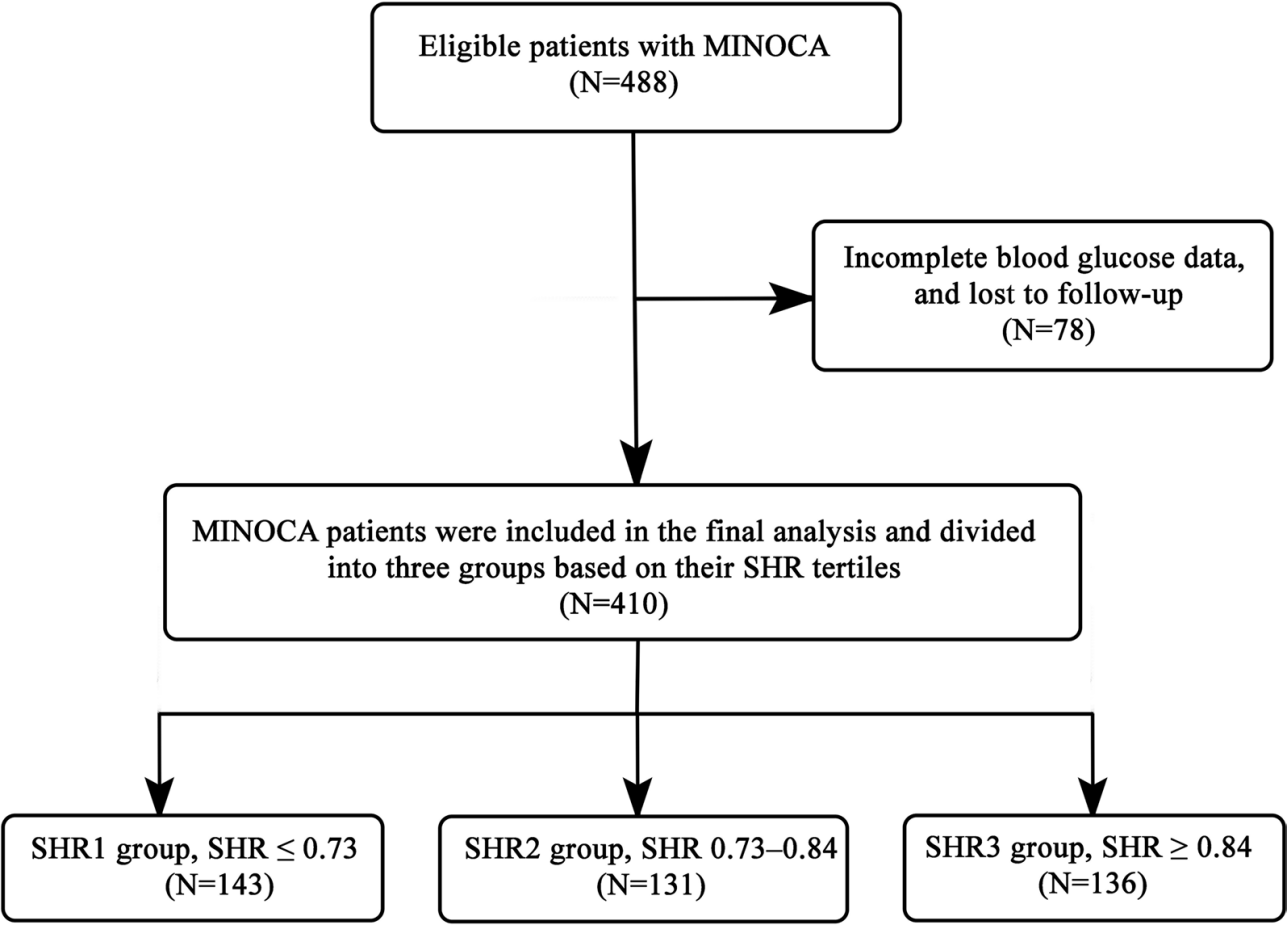
Flowchart of the study selection process. *MINOCA* myocardial infarction with non-obstructive coronary arteries, *SHR* stress hyperglycemia ratio

MINOCA was diagnosed in 488 consecutive patients, 78 of whom were lost to follow-up, did not have blood glucose data, and were excluded from the study. A total of 410 patients were included in the final analysis of the present study [216 (52.7%) were males; the mean age was 63.55 ± 13.81 years; and 79 (19.3%) had diabetes]. In this study, the patients were divided into three groups based on their SHR tertiles: [SHR1 group (SHR ≤ 0.73), (n = 143); SHR2 group (SHR 0.73–0.84), n = 131; and SHR3 group (SHR ≥ 0.84), n = 136] (Fig. 1).

Table 1 presents the baseline characteristics of the three groups. Patients in the SHR3 group had a higher rate of atrial fibrillation. The LVEF in the SHR3 group was lower than those in the SHR1 and SHR2 groups (52.73% vs. 55.68% and 55.78%, P = 0.041), whereas the left atrium size and left ventricular end-diastolic diameter were larger in the SHR3 group. Compared to the SHR1 and SHR2 groups, the SHR3 group had significantly higher serum cTnT, NT-proBNP, and CRP levels. However, no differences were observed between the three groups regarding other baseline characteristics or laboratory findings (all P > 0.05). The glucose-lowering medications for diabetic patients are shown in Additional file 1: Table S1.

### Clinical outcomes according to SHR tertiles and diabetes status

**Fig. 2:**
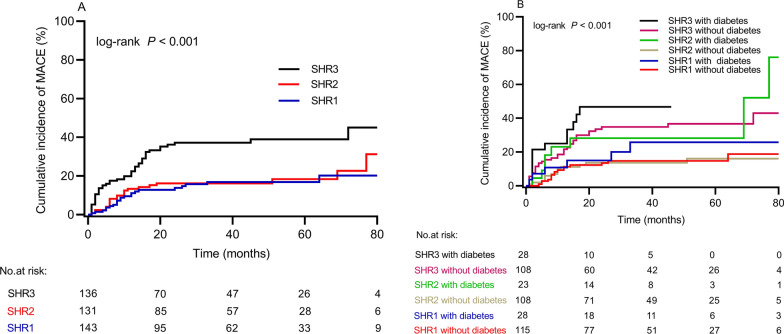
**(A)** Cumulative incidence of MACE based on the SHR tertiles; **(B)** Cumulative incidence of MACE based on the SHR tertiles and diabetes status. *MACE* major adverse cardiovascular events, *SHR* stress hyperglycemia ratio

A total of 92 patients (22.4%) developed MACE during the follow-up period. A significant increase in MACE was observed in the SHR3 group as compared to the SHR1 and SHR2 groups (35.3% vs. 15.4% and 16.8, respectively; P < 0.001) (Table 2). In Fig. 2A, the Kaplan–Meier curves demonstrate that SHR3 patients had the highest MACE risk compared to SHR1 and SHR2 patients (log-rank P < 0.001). For further analysis, the study population was divided into six subgroups based on SHR tertiles and diabetes status, including SHR1 with and without diabetes, SHR2 with and without diabetes, and SHR3 with and without diabetes. The results showed that those with SHR3 and diabetes had the highest hazard of MACE compared to other groups (log-rank P < 0.001) (Fig. 2B).

**Table 2:** Clinical outcomes according to different SHR tertiles

	SHR1 (≤ 0.73) N = 143	SHR2 (0.73–0.84) N = 131	SHR3 (≥ 0.84) N = 136	P value
MACE, n (%)	22 (15.4)	22 (16.8)	48 (35.3)	< 0.001
Cardiac death, n (%)	7 (4.9)	4 (3.1)	12 (8.8)	0.110
Non-fatal MI, n (%)	1 (0.7)	1 (0.8)	2 (1.5)	0.771
Heart failure, n (%)	3 (2.1)	4 (3.1)	8 (5.9)	0.220
Angina rehospitalization, n (%)	10 (7.0)	13 (9.9)	22 (16.2)	0.044
Stroke, n (%)	1 (0.7)	0	4 (2.9)	0.071

*MACE* major adverse cardiac events, *MI* acute myocardial infarction, *SHR* stress hyperglycemia ratio

### Predictive factors of MACE

The univariate and multivariate Cox regression analysis of MACE are shown in Tables 3,  4. Univariate Cox regression models showed that the SHR3 is associated with a 2.659-fold increased risk of MACE (HR 2.659; 95% CI 1.604–4.407, P < 0.001). Age, reduced LVEF, diabetes, atrial fibrillation, CK-MB, and NT-proBNP levels were also predictive factors of MACE in the univariate regression analysis.

**Table 3:** Univariate Cox regression analysis for endpoint events

	HR	95% CI	P-value
Age	1.024	1.008–1.040	0.004
Sex	0.960	0.638–1.447	0.847
BMI	1.009	0.936–1.087	0.817
LVEF	0.973	0.957–0.988	0.001
Hypertension	1.417	0.932–2.155	0.103
Diabetes	1.776	1.128–2.799	0.013
Hyperlipidaemia	1.065	0.592–1.916	0.834
Smoking	1.015	0.670–1.537	0.944
Atrial fibrillation	1.726	1.006–2.960	0.048
STEMI	1.063	0.692–1.634	0.780
Coronary stenosis	1.388	0.913–2.110	0.126
cTnT	1.036	0.974–1.103	0.263
Creatine kinase-MB	1.003	1.000–1.006	0.024
NT-proBNP	1.000	1.000-1000	0.003
TC	0.905	0.736–1.112	0.341
LDL-C	0.924	0.725–1.179	0.526
HDL-C	1.039	0.556–1.938	0.905
TG	1.131	0.956–1.337	0.152
CRP	1.052	0.859–1.288	0.621
SHR tertiles			
SHR1	Reference	Reference	
SHR2	1.133	0.628–2.047	0.677
SHR3	2.659	1.604–4.407	< 0.001

*BMI* body mass index, *LVEF* left ventricular ejection fraction, *STEMI* ST-segment elevation myocardial infarction, *cTnT* cardiac troponin, *NT-proBNP* N-terminal pro-brain natriuretic peptide, *TC* total cholesterol, *HDL-C* high density lipoprotein, *LDL-C* low-density lipoprotein, *TG* triglyceride, *CRP* C reactive protein, *SHR* stress hyperglycemia ratio, *HR* hazard ratio, *CI* confidence interval

**Table 4:** Multivariable cox regression analysis for endpoint events

	HR	95% CI	P-value
Age	1.016	0.999–1.034	0.063
LVEF	0.983	0.965–1.001	0.060
Diabetes	1.465	0.904–2.375	0.121
Atrial fibrillation	1.611	0.924–2.809	0.093
Creatine kinase-MB	1.004	1.001–1.007	0.012
NT-proBNP	1.000	1.000-1000	0.740
SHR3	2.465	1.461–4.159	0.001

*LVEF* left ventricular ejection fraction, *NT-proBNP* N-terminal pro-brain natriuretic peptide, *SHR* stress hyperglycemia ratio, *HR* hazard ratio, *CI* confidence interval

After excluding confounding factors with a P < 0.10 in the univariate analysis, multivariate Cox regression analysis showed that the SHR3 group remained associated with increased 2.465-fold of MACE (adjusted HR, 2.465; 95% CI 1.461–4.159, P = 0.001), along with CK-MB levels (adjusted HR, 1.004; 95% CI  1.001–1.007; P = 0.012).

### Optimal cut-off value of SHR for predicting outcomes among MINOCA

**Fig. 3:**
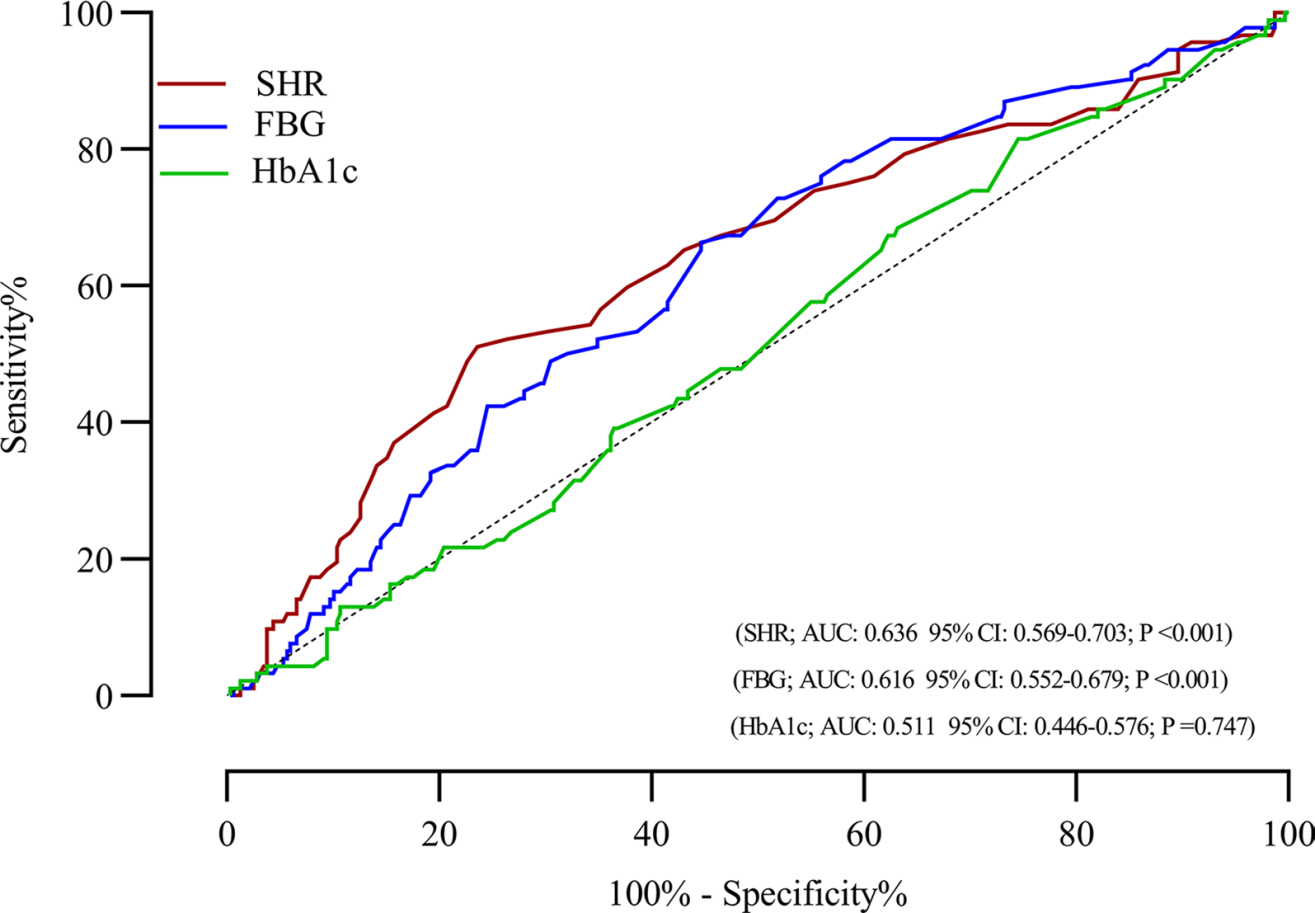
Receiver operating characteristic analysis of the ability of the SHR, FBG, and HbA1c to predict MACE in MINOCA patients. *AUC* area under the curve, *SHR* stress hyperglycemia ratio, *CI* confidence interval

The ROC curve of SHR, FBG, and HbA1c were displayed in Fig. 3 for the prediction of MACE among MINOCA patients, which demonstrated that SHR was consistently better predictor of MACE, with an AUC of 0.636 (95% CI  0.569–0.703; P < 0.001), while the AUC of FBG and HbA1c were (0.616 95% CI 0.552–0.679; P < 0.001 and 0.511 95% CI 0.446–0.576; P = 0.747, respectively). Notably, we obtained that the optimal SHR cut-off value for predicting clinical MACE was 0.86.

### Correlation between SHR and myocardial injury parameters

**Fig. 4:**
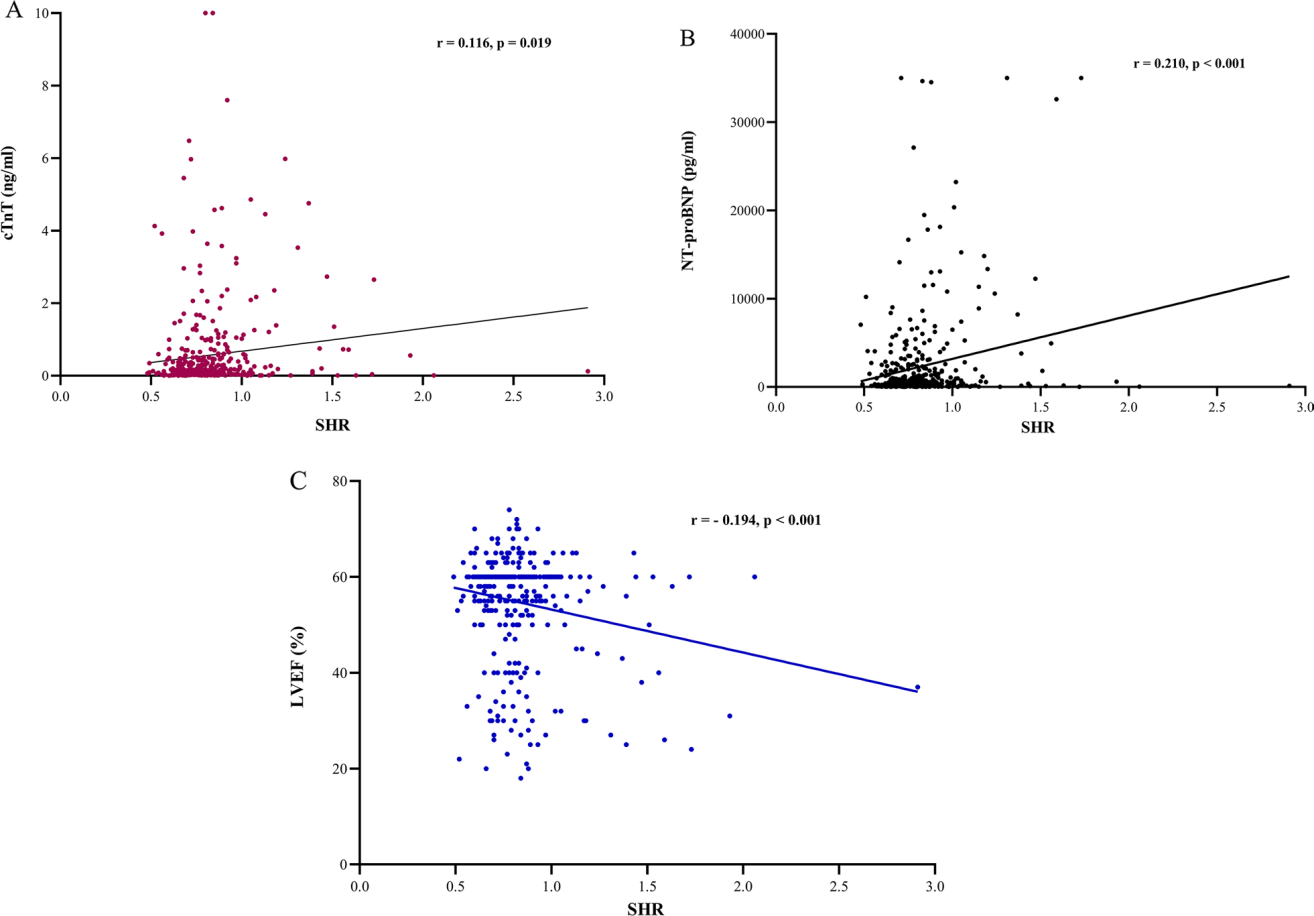
Correlation between SHR and cTnT **(A)**, NT-proBNP **(B)**, and LVEF **(C)**. *SHR* stress hyperglycemia ratio, *cTnT* cardiac troponin, *NT-proBNP* N-terminal pro-brain natriuretic peptide, *LVEF* left ventricular ejection fraction

The correlation between SHR and myocardial injury parameters, such as cTnT, CK-MB, NT-proBNP, and LVEF, was further examined. The results demonstrated that the SHR correlated well with cTnT, NT-proBNP, and LVEF among MINOCA patients (r = 0.116, r = 0.210, and r = − 0.194, respectively) (Fig. 4). However, SHR did not correlate with other myocardial injury parameters, such as creatine CK-MB (data not shown).

## Discussion

The present study, to our knowledge, is the first to evaluate the association between SHR and clinical outcomes among MINOCA patients. The novel findings of this study were: (1) a higher risk of clinical outcomes was observed for MINOCA patients with high SHR; (2) SHR was independently associated with long-term risk of MACE in patients suffering from MINOCA; (3) SHR cut-off value of 0.86 was able to identify the high-risk MINOCA patients, and (4) SHR correlated well with markers of myocardial injury, such as cTnT, NT-proBNP, and LVEF. These findings indicate that SHR may play a vital role in prioritizing patients and a robust biomarker to predict future MACE in the MINOCA population.

MINOCA has gained significant attention and has been added as a subtype of MI to the fourth global definition of MI [23]. MINOCA represents a challenging heterogeneous clinical syndrome where multiple aetiologies are causative with no optimal management therapy, and the prognosis in this high-risk patient group is far from benign [4, 25]. A recent Italian genetic study on early-onset MI demonstrated that MACE rates among MINOCA patients were 27.8% over a median follow-up of 19.9 years, which did not differ significantly from MACE rates among patients with obstructive coronary artery disease (CAD) [26]. The SWEDEHEART registry reported that 13.4% of MINOCA patients had an all-cause death, and 23.9% experienced a cardiovascular event during 4.1 years of follow-up [7]. In our investigation, we discovered that the MACE rate among MINOCA patients was 22.4% over a mean of 34 months of follow-up. This finding is comparable to those observed in previous clinical studies above showing a high incidence of MACE in the MINOCA population. Despite no apparent coronary stenosis in MINOCA patients, the risk of adverse events is not negligible, which indicates that MINOCA still afflicted potential harm and deserves the same level of significance as obstructive CAD. Data regarding clinical risk scores and predictors of adverse clinical outcomes in MINOCA populations is scarce. We and some other clinical investigations have documented the correlation between various factors, including cardiac troponin, age, sex, thyroid hormones, LVEF, metabolic syndrome, hyperglycemia, total bilirubin, creatinine, TC, and C-reactive protein with worse outcomes in MINOCA [14, 27–33]. Thus, it is necessary to perform rapid and accurate risk stratification using robust predictors beyond traditional clinical measures to identify potential factors associated with patient outcomes.

Stress hyperglycemia is frequent in AMI patients and negatively affects their prognosis, as well as being independently associated with higher mortality rates and greater infarct sizes [9–11]. A recent study showed that admission stress hyperglycemia was also common among MINOCA patients and significantly predicted both short- and long-term adverse outcomes, implying that hyperglycemia may contribute directly to myocardial damage [12]. However, stress hyperglycemia reflects the severity of an emergency and poor glucose control to some extent. Additionally, it may worsen acute cardiac illness in several ways, such as increasing endothelial dysfunction, decreasing platelet nitric oxide responsiveness, aggravating microvascular obstruction, and inducing further hyperglycemic-mediated vascular damage mechanisms [16]. The SHR is a novel marker of true acute hyperglycemia conditions and is associated with adverse outcomes in patients with AMI. SHR was first reported by Roberts et al. as an independent biological biomarker for clinical outcomes among patients with various clinical disorders [17]. A large cohort study in Asia found a correlation between SHR and early and late cardiac outcomes among ACS patients [16]. An analysis by Xu et al. found a significant association between SHR and in-hospital mortality in patients with CAD [18]. The SHR significantly predicted all-cause mortality among 5841 STEMI patients and 4105 NSTEMI patients after one year of fellow up [19]. A recent study also found that SHR predicts mortality and adverse events in STEMI patients, both in diabetes and non-diabetic patients [20]. The prospective, nationwide, multi-center China AMI registry results demonstrated a significant positive correlation between SHR and long-term death in patients with AMI [34]. Numerous clinical studies have also indicated a link between SHR and unfavorable outcomes in AMI patients [21, 22, 35, 36]. Pasquale et al. also found that SHR significantly increases the risk of rehospitalization among 2,874 patients with ischemia with non-obstructive coronary arteries [37]. Given that SHR was used as an indicator for predicting future clinical events in patients with cardiovascular disease, it is still unclear whether it can effectively predict the risk of clinical outcomes in the MINOCA population. Recently, we found that fasting blood glucose was associated with poor clinical outcomes among patients with MINOCA [14]. In this study, the sample size and follow-up period of MINOCA were expanded, and SHR was examined for the first time as a predictive factor for clinical outcomes among the MINOCA cohort. We demonstrated that the risk of adverse events was significantly higher in MINOCA patients with a high SHR group. Interestingly, after adjusting for age, sex, traditional cardiovascular risk factors, and other relevant biochemical parameters, multivariate cox regression analysis showed that high SHR remained significantly correlated with worse clinical outcomes. Our findings suggest that SHR may play a potential role in the cardiovascular risk stratification of the MINOCA population.

The underlying mechanisms that relate SHR to unfavorable outcomes in the MINOCA are not recognized. In our study, patients with high SHR levels were associated with worse baseline features, as demonstrated by a higher degree of myocardial injury (elevated cTnT and NT-proBNP) and reduced LVEF compared to patients with low SHR. We also found a significant correlation between SHR and NT-proBNP, cTnT, and LVEF, which may contribute to an increased risk of MACE to some extent. Several clinical studies have confirmed such results among MI patients, which reported that SHR had a significant correlation with myocardial injury as shown by high peak cTnT and peak CK-MB values and their association with the severity of CAD (assessed by the Gensini score and Syntax score) [10, 35, 38]. On the other hand, we found that inflammatory marker such as CRP was higher among the high SHR group, which may also be associated with some unfavorable outcomes. It has been demonstrated in previous MI studies that stress hyperglycemia leads to increase inflammation burden, ischemia-reperfusion damage, and endothelial dysfunction, which are all strongly associated with large infarct sizes and poor clinical outcomes [9, 39, 40]. Further studies should confirm this finding in larger MINOCA cohort and determine the underlying mechanisms of SHR in MINOCA.

The best cut-off value of SHR to predict clinical outcomes in CAD patients differs among studies. Among 19,929 patients with CAD, 0.741 was the best cut-off value for SHR to predict clinical outcomes [18]. An optimal SHR cut-off value of 0.78 predicted poor prognosis in ACS patients [16]. The optimal SHR cut-off value for predicting all-cause mortality at one year among diabetic STEMI patients was 1.68 and 1.51 among non-diabetic STEMI patients; however, the SHR for NSTEMI was 1.53 in diabetics and 1.27 in non-diabetics [19]. Among diabetic and non-diabetic AMI populations, Cui et al. found that 1.20 and 1.08 were optimal cut-off values for SHR in predicting 2-year mortality [34]. Luo et al. found that 1.24 and 1.14 were the optimal cut-off values for SHR in diabetics and non-diabetics with AMI, respectively [41]. Additionally, among elderly AMI patients, the optimal cut-off value of the SHR for predicting in-hospital outcomes was 1.20, while 1.32 for predicting in-hospital mortality [42]. However, a definite SHR cut-off value for predicting adverse events among MINOCA has not yet been studied. The present study found that the best optimal SHR threshold from the AUC for predicting clinical MACE in MINOCA was 0.86. It is necessary to conduct more large-scale prospective clinical studies in the MINOCA population to determine whether a cut-off value for SHR can accurately predict future poor clinical outcomes.

## Strengths and limitations

The strength of this study is that it is the first to evaluate the role of SHR and obtain its cut-off value in predicting poor clinical outcomes among MINOCA patients. The information provided by our study can be used by physicians to follow up on selected patients more closely, increase the intensity of their goal-directed medical treatment, control their risk factors, and improve the quality of life among patients with MINOCA. However, there are several limitations of our investigation that must be noted. First, the present study has a retrospective design with a small sample size; the validity of these findings requires further prospective multi-center studies. Second, the results demonstrated here were conducted on the Chinese MINOCA population; consequently, they may not generalize to other populations. Third, although SHR is linked to adverse outcomes even after adjusting for a few potential variables, the impact of unmeasured confounding variables cannot be removed entirely. Fourth, we did not consider other adverse outcomes, such as a reduction in left ventricular ejection fraction or infarction size, in addition to the association between SHR and MACE. In addition, retrospective observational nature of our study and lack of other inflammatory markers such as procalcitonin or systemic immune-inflammatory index limit us to speculate their effects on clinical outcomes. Lastly, our study lacks data regarding hypoglycemic therapy during the follow-up period; therefore, we are not able to assess their effect on the prognosis of patients.

## Conclusion

Our data indicate, for the first time, that SHR is independently associated with poor long-term prognosis in patients suffering from MINOCA. The optimal SHR cut-off value for predicting clinical MACE among MINOCA patients was 0.86. These findings suggest that SHR may play a potential role in the cardiovascular risk stratification of the MINOCA population.

## Supplementary information

**Additional file 1: Table S1.** Glucose-lowering therapy of diabetes patients. 

## Data Availability

The data analyzed in this study can be obtained from the corresponding author with a reasonable request.

## Abbreviations

CVD: Cardiovascular diseases
AMI: Acute myocardial infarction
MINOCA: Myocardial infarction with non-obstructive coronary arteries
MACE: Major adverse cardiac events
ACS: Acute coronary syndrome
SHR: Stress hyperglycemia ratio
LVEF: Left ventricular ejection fraction
HbA1c: Hemoglobin A1c
FBG: Fasting blood glucose
cTnT: Cardiac troponin
NT-proBNP: N-terminal pro-brain natriuretic peptide
TC: Total cholesterol
HDL-C: High-density lipoprotein
LDL-C: Low-density lipoprotein
TG: Triglyceride.
