# Supplementary material for: Association of stress hyperglycemia ratio and poor long-term prognosis in patients with myocardial infarction with non-obstructive coronary arteries
Source: Cardiovasc Diabetol. 2023 Jan 16;22:11. doi: 10.1186/s12933-023-01742-6 (PMC9843969; doi:10.1186/s12933-023-01742-6)
Supplement: Supplementary file 1 — Additional file 1: Table S1. Glucose-lowering therapy of diabetes patients. [file 12933_2023_1742_MOESM1_ESM.docx]

**Table S1. Glucose-lowering therapy of diabetes patients**

|  | Diabetes mellitus (n = 79) |
| --- | --- |
| Insulin, n (%) | 18 (22.8) |
| Metformin, n (%) | 25 (31.6) |
| Glinides, n (%) | 26 (32.9) |
| Alpha-glucosidase inhibitors, n (%) | 9 (11.4) |
| SGLT-2 inhibitors, n (%) | 3 (3.8) |

*SGLT-2 inhibitors* sodium glucose cotransporter-2 inhibitors.
